# Eye tracking in an everyday environment reveals the interpersonal distance that affords infant-parent gaze communication

**DOI:** 10.1038/s41598-019-46650-6

**Published:** 2019-07-17

**Authors:** Hiroki Yamamoto, Atsushi Sato, Shoji Itakura

**Affiliations:** 1 Graduate School of Letters, Kyoto University, Yoshida Honmachi, Sakyo-ku, Kyoto, 606-8501 Japan; 2 Faculty of Human Development, University of Toyama, 3190 Gofuku, Toyama, 930-8555 Japan; 3 Center for Baby Science, Doshisha University, 4-1-1 Kizugawadai, Kizugawa, 619-0225 Japan

**Keywords:** Social behaviour, Human behaviour

## Abstract

The unique morphology of human eyes enables gaze communication at various ranges of interpersonal distance. Although gaze communication contributes to infants’ social development, little is known about how infant-parent distance affects infants’ visual experience in daily gaze communication. The present study conducted longitudinal observations of infant-parent face-to-face interactions in the home environment as 5 infants aged from 10 to 15.5 months. Using head-mounted eye trackers worn by parents, we evaluated infants’ daily visual experience of 3138 eye contact scenes recorded from the infants’ second-person perspective. The results of a hierarchical Bayesian statistical analysis suggest that certain levels of interpersonal distance afforded smooth interaction with eye contact. Eye contacts were not likely to be exchanged when the infant and parent were too close or too far apart. The number of continuing eye contacts showed an inverse U-shaped pattern with interpersonal distance, regardless of whether the eye contact was initiated by the infant or the parent. However, the interpersonal distance was larger when the infant initiated the eye contact than when the parent initiated it, suggesting that interpersonal distance affects the infant’s and parent’s social look differently. Overall, the present study indicates that interpersonal distance modulates infant-parent gaze communication.

## Introduction

Human infants learn various types of knowledge, such as language, tool use, and cultural behaviour, from others in the first two years of life. Gaze communication, such as eye contact, gaze following, and joint attention, is the basis of infants’ social learning. It is well known that gaze communication contributes to infants’ social development in both the short term and the long term. Humans have a special sensitivity to direct eye contact from birth^1^. Eye contact modulates infants’ concurrent and immediately following cognitive processing or behavioural responses^2^ and enables fast and efficient social learning^3^. Gaze communication also longitudinally affects infants’ developmental trajectory by affecting important aspects such as language development^4^ and social skills^5^. Furthermore, deficits in eye contact are a hallmark of autism, and infants later diagnosed with autistic spectrum disorder (ASD) show a decreased level of eye contact from 2 to 6 months of age^6^. These studies indicate that gaze communication is important for shaping infants’ developmental trajectory. Because gaze communication strongly contributes to social development, many previous studies have focused on infants’ internal cognitive mechanisms that enable gaze communication in laboratory experiments.

However, in contrast to experimental conditions, in everyday life, infants face complex social contexts surrounded by a diverse array of objects with moving heads and eyes, complicating gaze communication. Moreover, events occurring during a social interaction are usually situated in the context of previous interactions. The moment-to-moment interests, goals, and behaviours of each participant in a social interaction are various and open-ended. Thus, to understand how infants and parents achieve gaze communication in such a messy environment, it is important to investigate not only the infant’s internal cognitive mechanism, which enables gaze communication in the experiment room, but also the ecological contexts in which gaze communication occurs in the infant’s everyday life and how the ecological context changes along with the infant’s development. Recently, by using head-mounted cameras and head-mounted eye trackers worn by infants, visual statistics from infants’ first-person perspective have been evaluated^7,8^.

Infants’ first-person visual experiences are determined by the disposition of the infant’s body in space^8^. Previous studies have reported that infants’ visual experiences during face-to-face interaction are affected by the posture, locomotor status and morphology of the infant’s body. Crawling infants see only the floor and must stop crawling and sit up to see social partners^9,10^. When infants interact with objects, manual action with their short arms often creates views in which a single object is visually dominant; this process is related to advances in object name learning^11,12^. Although these studies showed how infants’ visual experiences are affected by the infant’s body, little is known about how infants’ visual experiences in gaze communication are affected by the spatial disposition between the infant and the environment, for example, infant-parent distance.

Another important aspect of interpersonal distance is its variability, which characterises human gaze communication. Human eyes have a widely exposed white sclera surrounding the darker iris. Compared to other primate species, this unique morphology allows “gaze-signalling” and makes it easy to discern the direction in which humans are looking^13^. Furthermore, the morphology of human eyes also enables gaze communication from a large interpersonal distance. Humans can detect gaze signals over distances as far as 10 m^14^, and our gaze communication occurs at various ranges of interpersonal distance^15^. This contrasts with nonhuman primates, whose eye contact usually occurs at a relatively close distance^16^. Descriptions of gaze communication in the wild^17,18^ lead to the estimation that almost all such interactions occur at distances of less than 1 m. Although gaze communication at various ranges of distance characterises one aspect of human communication, there is no quantitative research about how interpersonal distance affects infants’ visual experiences in gaze communication.

Here, using head-mounted eye trackers, we explore infants’ visual experiences of gaze communication in everyday life from the parents’ first-person perspective, that is, the infants’ second-person perspective. A previous study has reported that a head-mounted camera worn by the infant’s social partner enables us to measure eye contact during a live social interaction more reliably and more validly than when using a stationary camera^19^. Recording gaze communication from the infant’s second-person perspective makes it possible to safely evaluate the statistical properties of a series of eye contacts between the infant and parent in their home environment. The purpose of this study is to evaluate the effect of infant-parent distance on daily gaze communication between the infant and parent. By recording daily gaze communication from the infant’s second-person perspective, the present study investigated (i) the number of eye contacts exchanged between the infant and parent in a series of eye contacts and (ii) the number of eye contacts for which the infant or parent initiates the contact in a series of eye contacts.

Human social interaction is held at various ranges of interpersonal distance. Hall^15^ categorised interpersonal distance into 4 distinct zones: intimate distance, personal distance, social distance and public distance. Personal distance is interpersonal distance for interactions among familiar friends or family, and the space inside the boundary of personal distance is well known as “personal space^20^”. Although human gaze communication occurs at various ranges of interpersonal distance, we predict that if gaze communication that occurs between an infant and a parent is held at personal distance, there may be an adequate interpersonal distance at which eye contact will be exchanged smoothly. If the interpersonal distance is too large or too small, eye contact interactions will not be exchanged continuously. Collectively, we predict that the number of eye contacts exchanged between the infant and parent will follow an inverse U-shaped pattern with interpersonal distance. In addition, if the infant and parent have their own preferred interpersonal distance for the social look, the number of eye contacts that the infant or parent initiates will also follow an inverse U-shaped pattern with interpersonal distance.

In addition to interpersonal distance, two factors may affect the number of eye contacts between the infant and parent in the home environment. The first is the infant’s age in months. As infants grow, they become more socially motivated and initiate social looking behaviour such as joint attention^5^. Initiating joint attention often accompanies infants’ use of gestures and eye contact^5^. As the infant’s age in months increases, the number of eye contacts that the infant initiates may increase, and the number of eye contacts exchanged between the infant and parent may also increase. The second factor is the infant’s locomotor status. The onset of walking is a developmental transition that sets in motion a cascade of change across a range of domains, including social interactions^21–24^. Previous studies comparing gaze behaviour between crawling infants and walking infants in the experiment room reported that a walking infant obtains higher and farther visual information in the environment^9^ and that an infant’s upright posture increases eye contacts between an infant and parent^10^. Such perceptual or behavioural change accompanied by the onset of walking may allow infants to initiate eye contacts and lead to an increase in the number of eye contacts exchanged between the infant and parent in their daily lives.

In this study, we investigated multiple factors that affect the infant’s daily gaze communication and evaluated which factors are the most predictive of the number of eye contacts exchanged and eye contacts that the infant or parent initiate with three potential factors: interpersonal distance, age in months and locomotor status.

## Methods

### Participants

**Figure 1:**
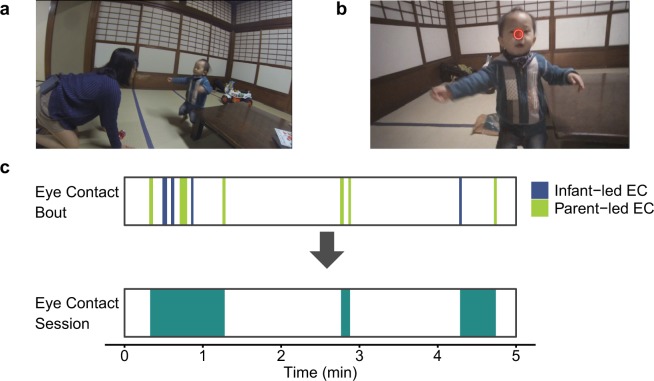
(**a**) We observed daily infant-parent interaction using head-mounted eye trackers worn by the parents. The infants and parents could move freely in their home environment. (**b**) Image from the scene camera of a head-mounted eye tracker worn by a parent. The red circle in the image represents the parent’s gaze fixation. Continuous images with infant gaze directed to the parent and the parent gaze on the infant’s face were defined as an eye contact bout (EC bout). (**c**) There were two types of EC bouts: infant-led EC bout (blue) and parent-led EC bouts (light green). We defined a series of EC bouts with short inter-eye-contact-bout intervals (IEI) as an eye contact session (EC session; green).

Five healthy, full-term infants (1 male and 4 females; A-E) and their mothers contributed data. All had normal or corrected-to-normal vision. Participants were recruited from infants registered in our laboratory database, which was constructed by delivering flyers and through the lab website. All participants were of Japanese ethnicity. Participants received a small monetary remuneration for their participation. All infants participated with written informed consent from their parents. In addition, for publication of identifying images in an online open-access publication, we obtained informed consent from parents of the infant, as shown in Fig. 1. This research was approved by the ethics review board at the Unit for Advanced Studies of the Human Mind, Kyoto University and was conducted in accordance with the Helsinki Declaration guidelines and regulations.

### Data collection

We visited each participant’s home and observed their social interaction on alternate weeks beginning when the infants were 10 to 15.5 months of age, an age range with which infants’ gaze behaviour begins to grow and walking generally emerges (see Supplementary Table S1). Certain home environment-related variables, such as house area and rearing style, may affect infants’ daily activities but are difficult to measure. To clearly detect the effect of the infant’s development, we chose to use a longitudinal design because the home environment-related variables above might be the same along each infant’s development.

The parents were not told that we were interested in the interpersonal distance or the timing of the eye contacts; instead, they were told that we were interested in the infants’ everyday activity. The parents were told that they could engage in any daily activities and that the parents and infants could go anywhere in their home and play with any of the available toys (Fig. 1a). We observed and recorded the infants’ and parents’ daily activity up to 1.5 h each day. Observation occurred when parents played with the infants; this play time was interrupted with small housekeeping tasks. We stopped video recording at the time of breastfeeding and restarted recording after breastfeeding. When the infant slept during the observation period, we stopped video recording at that time. After the observation, we measured the infant’s face size (between the chin and the eyebrows).

During the observation, the parent wore a head-mounted eye tracker (Tobii Gasses 2, Tobii Technology), which consisted of glasses, a wireless transmitter, and a battery pack. The glasses included an eye camera that records eye movements, a scene camera capturing the first-person view from the parent’s perspective, a microphone, a gyroscope and an accelerometer. The scene camera’s visual field was 90 degrees, providing a broad view but one less than the full visual field of the parents (approximately 180 degrees). The eye tracking system recorded the parent’s egocentric-view video at 25 Hz and gaze direction in that view at 50 Hz. The equipment was light and comfortable to wear, allowing the parent to move freely and to switch between postures with ease.

Before every recording, the parent wearing the head-mounted eye tracker was instructed to look at and focus on the centre of a card with a black and white target held at arm’s-length distance. Calibration was performed using eye-tracking software (Tobii Glasses Controller).

Although this research is a longitudinal observation study, we could not collect data when infant E was 12 months of age because infant E was in poor physical health. Data were collected from July 2015 to March 2016. The mean observation time for each day was 1 h 25 min, and the mean total observation time for one infant was 16 h 48 min.

### Data processing

#### Locomotor status

Throughout each infant’s entire observation period, we defined the observation day of the “acquisition of walking” using the parent’s perspective video. We categorised the infant’s locomotor status before the acquisition of walking as “crawler”; otherwise, we classified it as “walker” (see Supplementary Information).

#### Eye contact bout

We first identified the video frame of each eye contact from the parent’s perspective video frame by frame using the Tobii Glasses Analyzer software (Tobii Technology). The video frame of eye contact was defined as the video frame of the “parent’s looking at the infant’s face”, and the infant’s gaze was coded as “directed to parent” by the coders. The software (Tobii Glasses Analyzer) displayed the parent’s perspective video with the point of the parent’s gaze indicated on each video frame (Fig. 1b). The video frame of the “parent’s looking at infant’s face” was defined as the video frame at which the parent’s gaze was on the infant’s face, between the chin and the eyebrows. Coding whether the infant’s gaze was directed to the parent from the parent’s perspective video was based on Edmunds *et al*.^19^. Because meaningful eye contact should last some amount of time longer than a single video frame (40 ms) but should also be inclusive, as many meaningful participants look as long as possible (given their monitoring of the whole scene with frequent brief glances), eye contact bout (EC bout) was defined as continuous eye contact video frames that could include glances from either partner for less than 1 s but not greater than 1 s. The second coder independently judged 20% of the EC bouts, which were randomly selected from all EC bouts, with 96% intercoder agreement.

#### Initiator of eye contact bout

For each EC bout, either the parent or infant was the initiator who first looked at the partner before the EC bout. For each EC bout, by checking the parent’s perspective video backward from the first frame of EC bout, we determined which partner was first in time to meet our definition of eye contact and categorised the EC bout as either an infant-led EC bout or a parent-led EC bout. If the parent’s gaze was not on the infant’s face but the infant’s gaze was “directed to the parent” before the EC bout, the EC bout was coded as an infant-led EC bout. In the opposite case, the EC bout was coded as a parent-led EC bout. The second coder independently judged the initiator of eye contact from 20% of the EC bouts with 91% intercoder agreement.

#### Eye contact session

EC bouts occurred intermittently. It is not appropriate to treat each EC bout as an independent observation unit because it is expected that EC bouts that are close in time were similar to each other. We defined an eye contact session (EC session) as a series of EC bouts with short inter-eye-contact-bout intervals (IEIs) and use it as an independent observation unit (Fig. 1c). To determine the IEI criteria at which a new EC session begins by using a data-driven procedure, we performed statistical analysis based on the method described by Langton *et al*.^25^. Supposing that the distribution of IEIs follows a mixture of two gamma distributions representing IEIs within the same EC session and IEIs between adjacent EC sessions, EC bouts that occurred within 44.3 s of a former EC bout were defined as the same EC session (see Supplementary Information for how we defined EC session, Table S2, Fig. S2).

#### Interpersonal distance at the time of the eye contact bout

We calculated the interpersonal horizontal distance from the video frame of the EC bout. The monocular camera generates a one-to-one relationship between the object and the image. Using this principle, we can estimate the distance between the parent’s camera and the infant’s face from known parameters: the focal length of the camera, pixel size and the real size of the infant’s face. We output the video frame of the onset of each eye EC bout as an image file, measured the pixel size of the infant’s face (between the chin and the eyebrows) for each image file and estimated the direct distance between the infant and parent. Moreover, by calculating the angle of the face for a horizontal plane using accelerometer data from the head unit, we estimated the horizontal distance between the infant and parent at the timing of the eye contact. The accelerometer data were smoothed by applying a bidirectional second-order Butterworth low-pass filter. Finally, we calculated the mean interpersonal horizontal distance of EC bouts for each EC session. The programming codes used to estimate horizontal interpersonal distances were written using Python 2.7.13 (www. python.org). The second coder independently measured the pixel size of the infant’s face for 20% of the EC bouts, and the pixel size was strongly correlated (*r* = 0.96).

### Data analysis

We removed 1504 EC bouts because of an unsuitable setting for our purposes, difficulty in coding, and outliers. Finally, we analysed 1206 EC sessions, including a total of 3138 EC bouts (see Supplementary Information for details on removing data).

We conducted two statistical analyses using a hierarchical Bayesian model. The core of the hierarchical Bayesian model is a generalised linear mixed model (GLMM)^26^ used to estimate the effects of various factors on the response variable. Analysis 1 was intended to estimate the factors affecting how many times eye contacts were exchanged between infant and parent, and the response variable was the number of EC bouts in each EC session following a negative binomial distribution. Analysis 2 was intended to estimate the factors affecting how many times each member of the dyad initiated eye contact with their partner, and the response variable was the number of infant-led or parent-led EC bouts in the EC session following a poisson distribution.

To determine the most parsimonious model, we compared models using the widely applicable information criterion (WAIC)^27^. We selected the model with the smallest WAIC value as the best model among relevant candidate model sets. In Analysis 1, the explanatory variables (fixed effect) of the maximum model were *age* (in months: *β*_1_), *walker* (walker or crawler: *β*_2_), and *distance* (m: *β*_3_), *distance*^2^ (m^2^: *β*_4_). In addition to these explanatory variables, the maximum model used in Analysis 2 also included *initiator* (infant-led EC bout or parent-led EC bout: *β*_5_), $initiator\times age$ (*β*_6_), $initiator\times walker$ (*β*_7_), $initiator\times distance$ (*β*_8_) and $initiator\times distanc{e}^{2}$ (*β*_9_) as fixed effects (see Supplementary Table S3). To consider the individual difference between the dyad members and to correct for overdispersion, we set a random intercept and random slope in the models. We chose weakly informative priors for the hyperprior of some random effects because they help to stabilise parameter estimates in the case of a small group size^27^.

In each analysis, when the best model included the effect of distance squared, we can predict the mean response variable as a quadratic curve for interpersonal distance. When we could draw predictions as quadratic curves from the selected model, we calculated extremal values using MCMC samples.

Models were fitted using the Hamiltonian Monte Carlo engine Stan 2.17.0^28^, in R 3.5.0^29^. All iterations were set to 6000 and burn in samples were set to 1000, with the number of chains set to four. The values of Rhat for all parameters were below 1.1, indicating convergence across the four chains^27^.

## Results

Although there was an individual difference in motor development, all infants changed their locomotor status from crawler to walker over the longitudinal observation (see Supplementary Fig. S1).

### Number of eye contacts exchanged

The best model included the effect of age, distance, and distance squared (see Supplementary Table S4). Referring to the 95% credible interval of each effect’s parameter (Table 1), the age (*β*_1_ = 0.043; [0.014, 0.072]), the distance (*β*_3_ = 1.84; [1.36, 2.32]), and the distance squared (*β*_4_ = −0.796; [−1.05, −0.552]) were the most clearly detected fixed effects on the number of EC bouts exchanged in each EC session because the 95% credible interval did not include zero.

**Table 1:** The posterior distribution of the parameters of the best model.

Analysis	Variable	Parameter	EAP	2.5%	97.5%
Analysis 1	age	*β*_1_	0.043	0.014	0.072
	distance	*β*_3_	1.84	1.36	2.32
	distance^2^	*β*_4_	−0.796	−1.05	−0.552
	*D*	−*β*_3_/2*β*_4_	1.16	1.06	1.30
Analysis 2	age (parent)	*β*_1_	−0.037	−0.075	0.0006
	age (infant)	*β*_1_ + *β*_6_	0.111	0.073	0.149
	distance (parent)	*β*_3_	1.38	0.868	1.88
	distance (infant)	*β*_3_ + *β*_8_	2.32	1.79	2.84
	distance^2^	*β*_4_	−0.830	−1.09	−0.574
	initiator	*β*_5_	−2.41	−3.07	−1.75
	${D}_{({\rm{parent}})}$	−*β*_3_/2*β*_4_	0.829	0.699	0.949
	${D}_{({\rm{infant}})}$	−(*β*_3_ + *β*_8_)/2*β*_4_	1.41	1.26	1.62
	Δ*D*	−*β*_8_/2*β*_4_	0.578	0.401	0.833

The mean (EAP) and quantiles (2.5% and 97.5%) of posterior distribution are shown.

**Figure 2:**
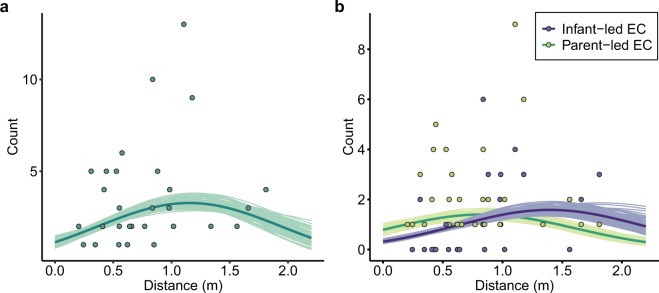
Relationship between the interpersonal distance and (**a**) the number of EC bouts in each session and (**b**) the number of infant-led EC bouts (blue) and parent-led EC bouts (light green) in each session on one observation day of one infant (when infant B was 11.5 months of age). The coloured dots represent the observed data. The darker lines represent the posterior mean of the mean number of EC bouts on the observation day. The lighter lines are 100 posterior samples.

Figure 2a shows the predictions for a representative observation day of one infant-parent dyad (see Supplementary Fig. S3). Because the distance squared effect (*β*_4_) had a negative value, the predictions of the number of EC bouts exchanged in each EC session were drawn as convex upward the quadratic curve. This result suggests that there was an interpersonal distance at which more eye contacts were exchanged with short intervals between infant and parent, and when the infant and parent were too close or too far away, eye contacts were less likely to be exchanged. The extremal value, where eye contacts were exchanged most between infant and parent, was in the range of 1.06–1.30 m (*D* = 1.16 m; Table 1).

The positive effect of age (*β*_1_) also suggests that the number of EC bouts exchanged in each session was likely to increase along the infant’s age in months.

### Number of infant-led and parent-led eye contacts

The best model included the effect of age, distance, distance squared, the initiator, the interaction between the initiator and age, and the interaction between the initiator and distance (see Supplementary Table S4). Referring to the 95% credible interval of each effect’s parameter (Table 1), the effect of age on infant-led EC bouts (*β*_1_ + *β*_6_ = 0.111; [0.073, 0.149]), the effect of distance on parent-led EC bouts (*β*_3_ = 1.38; [0.868, 1.88]), the effect of distance on infant-led EC bouts (*β*_3_ + *β*_8_ = 2.32; [1.79, 2.84]), the distance squared (*β*_4_ = −0.830; [−1.09, −0.574]), and the initiator (*β*_5_ = −2.41, [−3.07, −1.75]) were the most clearly detected fixed effects on the number of infant-led or parent-led EC bouts in each EC session because the 95% credible interval did not include zero. Because the selected model includes some interaction terms, the number of EC bouts in each EC session changed according to the infant’s age in months and interpersonal distance, as well as between infant-led EC bouts and parent-led EC bouts.

Figure 2b shows the predictions for a representative observation day of one infant-parent dyad (see Supplementary Fig. S4). Because the distance squared effect (*β*_4_) had a negative value, the predictions of the number of both infant-led and parent-led EC bouts in each EC session were drawn as convex upward quadratic curves. This result suggests that there was an interpersonal distance at which many infant-led EC bouts and parent-led EC bouts occurred. The extremal value for the parent-led EC bouts, where parent-led eye contacts occurred most, was in the range of 0.699–0.949 m (*D*_(parent)_ = 0.829 m). The extremal value for the infant-led EC bouts, where infant-led eye contacts occurred most, was in the range of 1.26–1.62 m (*D*_(infant)_ = 1.41 m), and the extremal value for the infant-led EC bouts was clearly farther than that of the parent-led EC bouts because their difference (Δ*D* = 0.578 m, [0.401, 0.833]) was a positive value. This result suggests that infant-led EC bouts were more likely to occur than parent-led EC bouts in each EC session when the infant and parent were located at a greater distance from each other. We also confirmed this tendency with additional analysis, where the response variable was a proportion of infant-led EC bouts in each EC session (see Supplementary Fig. S5 and Table S5).

The effect of age on infant-led EC bouts (*β*_1_ + *β*_6_) had a positive value. This result suggests that the number of infant-led EC bouts in each session was likely to increase along the infant’s age in months.

## Discussion

We evaluated the effect of infant-parent distance on gaze communication from two points of view: (i) how many times eye contacts were exchanged between infant and parent and (ii) how many times each member of the dyad initiated eye contact with their partner. We recorded daily social interaction from the infant’s second perspective and analysed the number of eye contacts exchanged between them and the number of eye contacts each member of the dyad initiated.

To estimate the factors affecting how many times eye contacts were exchanged between infant and parent, we analysed the number of eye contacts exchanged between them in a series of eye contacts in Analysis 1. Consistent with our prediction, the number of eye contacts exchanged between infant and parent changed in an inverse U-shaped manner along with interpersonal distance. That is, the eye contacts tended to occur less often when the infant and parent were too close or too far apart. This result indicates the existence of an interpersonal distance that affords smooth gaze communication between the infant and parent.

Hall^15^ defined interpersonal distance among familiar friends or family as “personal distance”. Although Hall^15^ noted that there is a cultural or situational variability in the space of “personal distance”, the actual interpersonal distance where eye contact was exchanged most continuously in this study (1.16 m) was located inside the personal distance previously defined by Hall^15^, which was 0.46–1.22 m. These results suggest that infant-parent distance modulates their live gaze communication and that the infant-parent distance where smooth gaze communication occurs is within personal distance.

The number of EC bouts exchanged in each session was likely to increase along the infant’s age in months. This result suggests that gaze communication between the infant and parent becomes exchanged more along the infant’s age in months in the first two years.

To estimate the factors affecting how many times each member of the dyad initiated eye contact with their partner, we investigated the number of eye contacts that the infant and parent initiated in a series of eye contacts in Analysis 2. Consistent with our prediction, regardless of whether the eye contact was initiated by the infant or parent, the number of eye contacts they initiated to social partner within session changed in an inverse U-shaped manner along with interpersonal distance. This result indicates that both the infants and parents have a preferred interpersonal distance that affords each of them to give a social look to their partner.

Gibson^30^ defined affordance as properties of the environment that are meaningful for animals, and properties such as size are meaningful only when an animal interacts with the environment at their ecological level. The inverse U-shaped tendency observed in this study may reflect the ecological level of interpersonal distance for social looks that is common to infants and parents. Interpersonal distance that is too close may change the emphasis of the modality of infant-parent interaction from vision to touch, and interpersonal distance that is too far away may make it difficult to detect the gaze signal of their partner.

Surprisingly, although the global effect of interpersonal distance was similar for both the infants and parents, the local characteristic of the interpersonal distance effect was different between them. The interpersonal distance where the infants initiated eye contacts most within session was greater than the distance where the parents initiated eye contacts most within session. This result suggests that interpersonal distance might afford social looking differently for infants and parents.

While parent-led EC bouts did not increase along the infant’s age in months, infant-led EC bouts increased along the infant’s age in months. A previous study has reported that as an infant grows, the infant becomes more socially motivated and initiates social looking behaviour such as joint attention^5^. Considering that gaze communication between infants and parents was exchanged more along the infant’s age in months in Analysis 1, the infant’s tendency to engage in a social look might have increased with age, and as a result, the number of eye contacts exchanged between infant and parent might also have increased.

Although both the infant and parent had a preferred interpersonal distance for initiating eye contact, it is unclear why the infant was likely to initiate eye contact more frequently from a farther distance than the parent. There may be a functional advantage of or need to engage in social looking from a farther distance for infants. Considering this view, we propose two hypotheses that are not mutually exclusive.

The first is related to the motor cost of looking behaviour. Looking is a holistic motor action that typically involves movements of the head, body and eyes^30^. When parents are out of the infants’ view, infants must move their heads and bodies to look at their parents’ face, and these motor actions require greater effort of the infants than merely moving their eyes. Although a previous study reported that the infant’s posture affects the motor cost of social looking^9,10^, infant-parent distance may also affect the motor costs of social looking. Usually, there is an eye height difference between the infant and parent, and infants have to tilt their heads up to look at their partners. When the infant and parent are too close, the infant’s motor cost to tilt his/her head up would become large because the degree to which he/she would have to crane his/her head upward becomes large. Thus, an adequately large (not too large) infant-parent distance may be preferable for infants to social look. The difference in the interpersonal distance where the infant and parent frequently initiate eye contact may come from such variability in the infant’s motor cost along interpersonal distance.

The second is related to joint attention. In goal-directed actions, the hands and eyes of the actor are tightly coordinated both temporally and spatially. When infants look at the actor’s manual engagement with objects, the hand movements and eye movements provide redundant information about where the actors are looking. Some studies that use a free-play context with a fixed infant-parent distance of 60–70 cm have reported that the infants achieved joint attention not with gaze following but with hand following^31–33^. Such an interpersonal distance (60–70 cm) may allow both the infant and parent to manually act on any objects between them. However, when interpersonal distance becomes large, there may be a space where each of the dyads cannot manually access the objects between them. If there are any objects in this space, hand-following may not be a sufficient solution to achieve joint attention because the redundancy of the hand and eye direction of an individual are no longer guaranteed with such a diverse array of objects. Although hand following is a cheap and effective solution to achieve joint attention in some contexts, gaze following may also be an effective solution in some complex contexts, and an adequately large interpersonal distance may afford social looking by infants.

Gibson^30^ indicated that different animals can perceive different affordances in the same environment. In our study, the infant and parent might perceive similar but different affordances for social looking from the same interpersonal distance. Although the motor cost or joint attention may be related to different social looking between infants and parents in the same interpersonal distance, it is difficult to test the hypotheses above from our data. Unless the infant’s first-person perspective is recorded, we cannot make inferences about joint attention or the motor cost on the infant’s social looking. Further study using recordings from the infant’s first-person perspective is needed to investigate why the infant is likely to social look from a farther distance than the parent.

The result that infant-parent distance affects gaze communication provides new insight regarding the previous challenge of evaluating infants’ visual experiences from the infant’s first-person perspective. Although infants’ visual experiences are determined by the disposition of the infants’ body in space^8^, many studies using head-mounted eye trackers for infants did not focus their research interests on the effect of the spatial disposition between the infant and the environment, including other people, but rather on the effect of the infant’s own posture, locomotor status^9,10^ or the morphology of the infant’s body^11,12^ on the infant’s first-person experience. Although this study did not record the infant’s first-person perspective with a head-mounted camera or head-mounted eye tracker, we can infer some aspects of the infant’s visual experience of gaze communication because when eye contact occurs between the infant and parent, both members of the dyad are “seeing” and “being seen”. This study is the first to investigate the effects of interpersonal distance on one aspect of infants’ visual experiences in live social interaction.

This study is also the first to investigate infant-parent gaze communication in their home environment using head-mounted eye trackers. To evaluate the infant’s visual experience not in an experiment room but in the home environment, some previous research used head-mounted cameras and recorded the infant’s first-person perspective visual scene^34–37^. Although infants and adults typically turn their heads and eyes in the same direction to attend to a visual event^38^, there is a methodological constraint to investigating gaze communication between infants and parents by using head-mounted cameras because the head and eyes do not always move together. Recording from the infant’s second-person perspective is a safe and low-cost solution to infer the dynamics of gaze communication, such as eye contact, in their everyday life.

This study also provides ecological validity to the previous results on infants’ social look. Previous studies reported that infants rarely look at their parent’s face^10,31–33,39^. Some of these studies used head-mounted eye trackers and recorded infant-parent interaction with a fixed distance of 60–70 cm^31–33^. The infant-parent distance of 60–70 cm is the distance where parent-led eye contact was more likely to occur than infant-led eye contact in our study and is also the distance at which the infant was less likely to social look than the parent. The characteristic of the interpersonal distance’s effect on the infant’s and parent’s social looking in this study provides ecological validity for the tendency of infants to rarely look at their parent, as noted in prior research. Moreover, it also provides a new prediction: that the infant may look at the parent more frequently when the interpersonal distance is adequately large (not too large). To test this prediction, it is necessary to compare gaze communication with various infant-parent distances by using head-mounted eye trackers on both of them.

One limitation of this study is its generalisability. Establishing the generality of our results will require more evidence because our data came from only five dyads. Moreover, because the result of our study is correlational and descriptive, an experimental test is needed to determine the difference in the interpersonal distance effect on social looking between infants and parents. In addition, there is another limitation to our method: the recordings are made from the infant’s second-person perspective. Although we detected eye contact between the infant and parent from the parent’s first-person perspective, eye contact is just one aspect of various kinds of gaze communication. Whether the effect of infant-parent distance found in this study can be generalised to other kinds of gaze communication, such as gaze following, joint attention and shared attention, remains unclear. Overall, in future research, it is necessary to record gaze communication by using head-mounted eye trackers on both infants and parents.

Although there are some limitations to this study, recording from the infant’s second-person perspective enabled us to investigate the ecological contexts in which gaze communication occurs in infants’ everyday lives. Taking advantage of the infant’s second-person perspective to record a free-moving dyad’s daily social interaction longitudinally, we evaluated the effect of interpersonal distance on infant-parent gaze communication. Although some theorists have emphasised the importance of situating infants in social interaction to understand social cognition^40,41^, the ecological characteristics of daily infant-parent interaction are not yet fully understood. Recording from the infant’s second-person perspective may play a complementary role in recording the infant’s first-person perspective and may be useful for investigating how infants’ daily gaze communication shapes their social cognition in the future.

## Supplementary information

Supplementary Information

## Data Availability

The datasets and the codes used for the models and graphs are available in the GitHub repository, https://github.com/dororo1225/GazeCommunication1.
