# Supplementary material for: Eye tracking in an everyday environment reveals the interpersonal distance that affords infant-parent gaze communication
Source: Sci Rep. 2019 Jul 17;9:10352. doi: 10.1038/s41598-019-46650-6 (PMC6637119; doi:10.1038/s41598-019-46650-6)
Supplement: Supplementary file 1 — Supplementary Information [file 41598_2019_46650_MOESM1_ESM.pdf]

Eye tracking in an everyday environment reveals the interpersonal distance that affords infant-parent gaze communication

***Hiroki Yamamoto[1], Atsushi Sato[2] and Shoji Itakura[1,3]**

[1]Graduate School of Letters, Kyoto University, Japan
[2]Faculty of Human Development, University of Toyama, Japan
[3]Center for Baby Science, Doshisha University, Japan

* Corresponding author:E-mail: yamamoto.hiroki.nr@gmail.com

Data Processing

Locomotor Status

The infants' behavioural data were coded from the respective parents' video for each observation day. We identified 3 types of infant movements – crawling, cruising, and walking – and recorded each behaviour with one-zero sampling [1] for 15 s. Crawling was defined as a series of steps with a prone posture, and infants were on hands and knees or hands and feet. Cruising was defined as a series of steps with an upright posture and supported (by a caregiver or furniture). Walking was defined as a series of steps with an upright posture and unsupported. Steps were defined in terms of alternating leg movements that changed the infants location on the floor. Steps could be omnidirectional (infants often stepped backward or sideways or in place). These definitions are based on those in Adolph *et al.* [2]. The second coder independently judged 20 % of the parent's perspective video with 92 % intercoder agreement.

Throughout each infant's entire observation period, the first observation day when the percentage of walking became larger than that of crawling was defined as "acquisition of walking", and we categorised the infant's locomotor status before acquisition of walking as "crawler" and otherwise as "walker" (Fig. S1).

To assess whether infant age was related to locomotor status, we set locomotor status on each observation day as the response variable and infant age as the explanatory variable in a generalised linear mixed model with binomial error structure. To consider individual differences, we set infant identity as a random intercept. The effect of infant age was tested with the statistical packages lme4 [3] and car [4] in R 3.5.0 [5]. The likelihood ratio test revealed a significant effect of infant age ($\chi^2(1) = 362.4, p < 0.0001$) and that as infants grew, their locomotor status was likely to be "walker".

Eye Contact Session

We defined eye contact session (EC session) as a series of eye contact bouts (EC bouts) with short inter-eye-contact-bout intervals (IEIs) and use it as an independent observation unit. To determine the IEI criteria for which a new EC session starts, we performed parameter estimation using a statistical model based on the method described by Langton *et al.* [6]. We applied a single gamma distribution model and a 2-process gamma distribution model and evaluated which model was more parsimonious based on widely applicable information criterion (WAIC) [7].

First, let us assume that the frequency of the observed IEIs x follows the single gamma distribution of shape parameter α_1 and rate parameter β_1. The probability density of the IEIs x is defined as

$$f(x; \alpha_1, \beta_1) = \frac{\beta_1^{\alpha_1} x^{\alpha_1 - 1} e^{-\beta_1 x}}{\Gamma(\alpha_1)} \tag{1}$$

where $\Gamma(\alpha_1)$ is the gamma function.

Next, let us assume that the frequency of the observed IEIs x follows the mixture of two gamma distribution components. One gamma distribution component of the shape parameter α_1 and rate parameter β_1 represents the IEIs in each EC session, and the other gamma distribution component of the shape parameter α_2 and rate parameter β_2 represents the IEIs between the adjacent EC sessions. The probability density of the IEIs x is defined as

$$f(x; \alpha_1, \beta_1, \alpha_2, \beta_2, p_1) = p_1 * \frac{\beta_1^{\alpha_1} x^{\alpha_1 - 1} e^{-\beta_1 x}}{\Gamma(\alpha_1)} + (1 - p_1) * \frac{\beta_2^{\alpha_2} x^{\alpha_2 - 1} e^{-\beta_2 x}}{\Gamma(\alpha_2)} \tag{2}$$

where $\Gamma(\alpha_1)$ and $\Gamma(\alpha_2)$ are the gamma functions. p_1 is the mixture ratio of the gamma distribution component of the shape parameter α_1 and rate parameter β_1.

The models were fitted using the Hamiltonian Monte Carlo engine Stan 2.17.0 [8], in R 3.5.0 [5]. All iterations were set to 5500 and burn in samples were set to 500, with the number of chains set to four. The value of Rhat for all parameters was below 1.1, indicating convergence across the four chains [9].

The best-performing model was the mixture of two gamma distribution model (Table S2). One gamma distribution component represented the IEIs in each EC session (α_1 = 1.02; β_1 = 0.061), and the other gamma distribution component represented the IEIs between adjacent EC sessions (α_2 = 0.806; β_2 = 0.007).

We set the IEI criteria at the intersection of the two estimated gamma distributions, that is, where the 2 processes occurred with the same probability, 44.3 s (Fig. S2). Then, the EC session was defined as continuous EC bouts that include IEIs briefer than 44.3 s.

Removal of Data

The purpose of this study was to evaluate how infant-parent distance affects their gaze communication in their everyday life. To evaluate eye contact when each member of the dyad was in a natural spatial location, we excluded EC bouts for which the infant's movements were constrained by the parent or environmental objects such as enclosure. For example, infants were sometimes put in play pens when the parent did not want to be disrupted by infants in order to do light housekeeping. Moreover, infants were sometimes held or carried by their parents in social interactions. We excluded EC bouts in such situations because infants could not adjust the interpersonal distance. Moreover, to detect the effect of locomotor status clearly, we try to control for the effect of the parent's posture and also excluded EC bouts when the parent did not sit on the floor because a previous study reported that when parents are standing or walking, infants' social look decreases more than when parents are sitting on the floor [10]. Thus, 18.3% of the total EC bouts were excluded.

Several EC bouts were also excluded from the analysis because we could not perform coding. A reason why we could not perform coding was because the interpersonal distance of some EC bouts was too close and the infant's face became larger than the visual angle of the scene camera. We also could not code the initiator of some EC bouts because they occurred with the infant and parent looking at each other's faces simultaneously. Thus, 12.8% of the total EC bouts were excluded.

Moreover, obvious outlier EC sessions were excluded from the dataset. The 3 SD of the distance of the EC sessions (mean distance of EC bouts in the same EC session) was calculated, and if the distance of the EC session exceeded 3 SD from the mean value, this EC session was marked as an outlier. Thus, 2.1% of the total EC sessions (including 1.2% of total EC bouts) were excluded.

For these reasons, we removed 1504 EC bouts and finally analysed 1206 EC sessions, including a total of 3138 EC bouts.

Data Analysis

We conducted two main statistical analyses and one additional analysis using hierarchical Bayesian models. The core of the hierarchical Bayesian model is a generalised linear mixed model (GLMM) [11] that estimates the effects of the various factors on the response variable. Analysis 1 was intended to estimate the factors affecting how many times gaze communication was exchanged between the infant and parent, and the response variable was the number of EC bouts exchanged in each EC session. Analysis 2 was intended to estimate the factors affecting how many times each member of the dyad initiated eye contact with their

partner, and the response variable was the number of infant-led or parent-led EC bouts in each EC session. Analysis 3 was intended to estimate the factors affecting the ratio of eye contacts initiated by the infant to those initiated by the parent, and the response variable was the proportion of infant-led EC bouts in each EC session.

To determine the most parsimonious model, we compared models using widely applicable information criterion (WAIC) [7]. In each model selection procedure, we included all likely explanatory variables and then excluded the explanatory variable and calculated the WAIC sequentially. Finally, we selected the model with the smallest WAIC value as the best model among relevant candidate model sets.

Models were fitted using the Hamiltonian Monte Carlo engine Stan 2.17.0 [8], in R 3.5.0 [5]. All iterations were set to 6000 and burn in samples were set to 1000, with the number of chains set to four. The values of Rhat for all parameters were below 1.1, indicating convergence across the four chains [9]. We chose conservative, weakly informative priors for the hyperprior of some random effects. This made our models sceptical of large effects and helped ensure convergence.

Analysis 1: Number of Eye Contact Bouts Exchanged

The response variable of the statistical model was the number of EC bouts exchanged in each EC session. A series of models with up to four explanatory variables (fixed effects; Table S3) were fitted, and the most parsimonious model was determined based on the WAIC.

First, let us assume that the observed number of EC bouts exchanged in each EC session k, that is, N_k, between the infant-parent dyad i on observation day j follows a negative binomial distribution of the mean μ_k, where μ_k represents the mean number of EC bouts exchanged in each EC session. The log link function is applied for μ_k such that the factors in the linear predictor affect μ_k multiplicatively. The linear predictor of the maximum model is defined as

$$\log \mu_k = \beta_0 + r_i + \beta_1 * \text{age}_{ij} + \beta_2 * \text{walker}_{ij} + \beta_3 * \text{distance}_k + \beta_4 * \text{distance}_k{}^2 \quad (3)$$

β_0 is the intercept, and the set of β_* from β_1 to β_4 represents the coefficients of explanatory variables (fixed effects). The explanatory variable age_{ij} represents the age in months of the infant i on observation day j, and the explanatory variable walker_{ij} indicates whether the locomotor status of the infant i was walker or not on observation day j. The explanatory variable distance_k represents the interpersonal distance of the eye contact session k. To consider differences in the dyad, we set the dyad identity as a random intercept r_i. We chose conservative, weakly informative priors for the hyperprior of random effect r_i.

When the best model includes the effect of interpersonal distance squared, we can predict the mean number of EC bouts as a quadratic curve for interpersonal distance. When we were able to draw predictions as quadratic curves from the selected model, we calculated the extremal value D using the MCMC samples.

Analysis 2: Number of Infant-led and Parent-led Eye Contact Bouts

The response variable of the statistical model was the number of infant-led or parent-led EC bouts in each EC session. A series of models with up to nine explanatory variables (fixed effects; Table S3) were fitted, and the most parsimonious model was determined based on the WAIC.

First, let us assume that the observed number of infant-led or parent-led EC bouts in each EC session k, that is, N_k, between the infant-parent dyad i on observation day j follows a poisson distribution of the mean λ_k, where λ_k represents the mean number of infant-led or parent-led EC bouts in each EC session. The

log link function is applied for λ_k such that the factors in the linear predictor affect λ_k multiplicatively. The linear predictor of the maximum model is defined as

$$\begin{aligned}\log \lambda_k = &\beta_0 + r_{i1} + \beta_1 * \text{age}_{ij} + \beta_2 * \text{walker}_{ij} + \beta_3 * \text{distance}_k + \beta_4 * \text{distance}_k{}^2 \\ &+ (\beta_5 + r_{i2} + \beta_6 * \text{age}_{ij} + \beta_7 * \text{walker}_{ij} + \beta_8 * \text{distance}_k + \beta_9 * \text{distance}_k{}^2) * \text{initiator}_k \\ &+ r_k\end{aligned} \tag{4}$$

β_0 is the intercept, and the set of β_* from β_1 to β_9 represents the coefficients of the explanatory variables (fixed effects). The explanatory variables age_{ij}, walker_{ij} and distance_k represent the same variable as the statistical model of Analysis 1. The explanatory variable initiator_k is a dummy variable that indicates whether N_k is the number of infant-led EC bouts or parent-led EC bouts. To consider the individual differences of the infant and parent separately, we set dyad identity as the random intercept r_{i1} and random slope r_{i2} in the fixed effect *initiator*. We also set eye contact session identity as the random intercept r_k to correct for overdispersion. We chose conservative, weakly informative priors for the hyperprior of the random effects, r_{i1} and r_{i2}.

If $\lambda_{k(\text{parent})}$ is the mean number of parent-led EC bouts in each EC session k, the linear predictor of the maximum model is defined as

$$\log \lambda_{k(\text{parent})} = \beta_0 + r_{i1} + \beta_1 * \text{age}_{ij} + \beta_2 * \text{walker}_{ij} + \beta_3 * \text{distance}_k + \beta_4 * \text{distance}_k{}^2 + r_k \tag{5}$$

If $\lambda_{k(\text{infant})}$ is the mean number of infant-led EC bouts in each EC session k, the linear predictor of the maximum model is defined as

$$\begin{aligned}\log \lambda_{k(\text{infant})} = &\beta_0 + \beta_5 + r_{i1} + r_{i2} + (\beta_1 + \beta_6) * \text{age}_{ij} + (\beta_2 + \beta_7) * \text{walker}_{ij} \\ &+ (\beta_3 + \beta_8) * \text{distance}_k + (\beta_4 + \beta_9) * \text{distance}_k{}^2 + r_k\end{aligned} \tag{6}$$

When the best model includes the effect of interpersonal distance squared and any interaction effect, we can draw the predictions of the mean number of infant-led EC bouts and parent-led EC bouts as two different quadratic curves for interpersonal distance. When we were able to draw two different predictive curves from the selected model, we calculated the extremal value for each quadratic curve using MCMC samples. The extremal value of the infant-led EC bout's predictive curve was defined as $D_{(\text{infant})}$, and the extremal value of the parent-led EC bout's predictive curve was defined as $D_{(\text{parent})}$. We took the difference of two extremal values ΔD as follows:

$$\Delta D = D_{(\text{infant})} - D_{(\text{parent})} \tag{7}$$

Analysis 3: Proportion of Infant-led Eye Contact Bouts

In the result of Analysis 2, the actual characteristic of the interpersonal distance effect was different for the infant-led EC bouts and the parent-led EC bouts. The interpersonal distance where infant-led eye contact occurred most was larger than that of parent-led eye contacts. To confirm this tendency, we conducted an additional analysis in which the response variable was the proportion of infant-led EC bouts in each EC session (Analysis 3).

Supplementary Methods

The response variable of the statistical model was the proportion of infant-led eye contact bouts in each EC session. A series of models with up to four explanatory variables (fixed effects; Table S3) were fitted, and the most parsimonious model was determined based on the WAIC.

First, let us assume that the observed number of infant-led EC bouts Y_k in the total number of EC bouts N_k at EC session k between the infant-parent dyad i on observation day j follows a binomial distribution of parameter q_k, the ratio of infant-led EC bouts in each EC session. The logit link function is applied for q_k. The linear predictor of the maximum model is defined as

$$\log \frac{q_k}{1-q_k} = \beta_0 + r_i + \beta_1 * \text{age}_{ij} + \beta_2 * \text{walker}_{ij} + \beta_3 * \text{distance}_k + \beta_4 * \text{distance}_k{}^2 + r_k \quad (8)$$

β_0 is the intercept, and r_k is the random intercept for EC session k. To consider dyad differences, we set dyad identity as the random intercept r_i. The set of β_* from β_1 to β_4 represents the coefficients of the explanatory variables (fixed effects). The explanatory variables age$_{ij}$, walker$_{ij}$ and distance$_k$ represent the same variables as used in the statistical model in Analysis 1. We chose conservative, weakly informative priors for the hyperprior of the random effect r_i.

Supplementary Results

The best model included the effect of age in months and interpersonal distance (Table S4). Referring to the 95% credible interval of each effect's parameter (Table S5), the age in months (β_1 = 0.166; [0.115, 0.218]) and the interpersonal distance (β_3 = 1.06; [0.821, 1.32]) were the most clearly detected fixed effects on the proportion of infant-led EC bouts in each EC session because the 95% credible interval did not include zero.

Fig. S5 shows the predictions of the proportion of infant-led EC bouts in each EC session of each observation day. Because the interpersonal distance effect (β_3) had a positive value, the predictions of the proportion of infant-led EC bouts in each EC session increase along with interpersonal distance. This result suggests that infant-parent distance affects each member of the dyad's social look in gaze communication and the ratio of the infant's social look to the parent's social look.

The positive effect of age in months (β_1) also suggests that the proportion of infant-led EC bouts in each EC session was likely to increase along the infant's age in months. This result is consistent with the result that the EC bouts that were not parent-led but were infant-led increased along the infant's age in months in Analysis 2.

References

[1] Altmann, J. Observational Study of Behavior: Sampling Methods. *Behaviour* **49**, 227-266 (1974).

[2] Adolph, K. E. *et al.* How Do You Learn to Walk? Thousands of Steps and Dozens of Falls per Day. *Psychol Sci.* **23**, 1387-1394 (2012).

[3] Bates, D., Mächler, M., Bolker, B. & Walker, S. Fitting Linear Mixed-Effects Models Using lme4. *J Stat Softw.* **67**, (2015).

[4] Fox, J. & Weisberg, S. *An R Companion to Applied Regression* (Thousand Oaks CA: Sage, 2011), 2nd edn.

[5] R Core Team. *R: A Language and Environment for Statistical Computing*. R Foundation for Statistical Computing, Vienna, Austria (2018).

[6] Langton, S. D., Collett, D. & Sibly, R. M. Splitting Behaviour Into Bouts; a Maximum Likelihood Approach. *Behaviour* **132**, 781-799 (1995).

[7] Gelman, A., Hwang, J. & Vehtari, A. Understanding predictive information criteria for Bayesian models. *Stat. Comput.* **24**, 997-1016 (2013).

[8] Stan Development Team. RStan: the R interface to Stan (2018).

[9] Gelman, A. *et al*. *Bayesian data analysis* (Chapman & Hall/CRC, London, 2013), 3rd edn.

[10] Franchak, J. M., Kretch, K. S. & Adolph, K. E. See and be seen: Infant-caregiver social looking during locomotor free play. *Dev. Sci.* **21**, e12626 (2017).

[11] Crawley, M. J. *Statistics: an introduction using R* (Wiley, West Sussex, 2005).

Table S1. The number of infant-parent dyads and summary statistics of age in days for each observation day.

Age (months)	N	Mean	SD	Min.	Max.
10.0	5	309.8	2.32	307	312
10.5	5	326.8	2.64	322	329
11.0	5	340.6	2.15	338	344
11.5	5	354.0	3.58	349	359
12.0	4	371.5	3.35	368	377
12.5	5	384.6	3.07	382	390
13.0	5	403.4	5.39	396	411
13.5	5	417.2	4.26	413	424
14.0	5	432.4	3.32	427	437
14.5	5	448.4	3.67	445	455
15.0	5	465.8	4.12	461	471
15.5	5	481.2	2.79	476	484

Table S2. Widely applicable information criterion (WAIC), posterior mean (Posterior Mean), and posterior standard deviation (Posterior SD) for the evaluated two models for inter-eye-contact-bout intervals.

Model	WAIC (SE)	Posterior Mean (Posterior SD)				
		α_1	β_1	α_2	β_2	p_1
Mixture Gamma	44149.1 (216.8)	1.02 (0.041)	0.061 (0.006)	0.806 (0.041)	0.007 (0.0004)	0.571 (0.029)
Simple Gamma	44807.5 (215.5)	0.579 (0.010)	0.010 (0.0002)			

Table S3. Description of the fixed effects of the explanatory variables for the maximum model in Analyses 1 to 3. The "coefficient" column shows the symbols of the coefficients corresponding to the variables. All likely explanatory variables in each analysis are shown as "+", and "-" indicates the explanatory variables that are not taken into account in the analysis.

Variable	Description	Coefficient	Analysis 1	Analysis 2	Analysis 3
age	continuous<br>(in months; 10-15.5)	β_1	+	+	+
walker	categorical<br>(locomotor status; walker-crawler)	β_2	+	+	+
distance	continuous<br>(in meters; interpersonal distance)	β_3	+	+	+
distance^2	continuous<br>(interpersonal distance squared)	β_4	+	+	+
initiator	categorical<br>(infant-led EC bout - parent-led EC bout)	β_5	−	+	−
initiator : age	continuous<br>(0 or 10-15.5)	β_6	−	+	−
initiator : walker	categorical<br>(0 or walker-crawler)	β_7	−	+	−
initiator : distance	continuous<br>(0 or interpersonal distance)	β_8	−	+	−
initiator : distance^2	continuous<br>(0 or interpersonal distance squared)	β_9	−	+	−

Table S4. Widely applicable information criterion (WAIC), posterior mean (Posterior Mean), and posterior standard deviation (Posterior SD) for best 3 models in Analyses 1 to 3.

Model rank	WAIC (SE)	Posterior Mean (Posterior SD)								
		age β_1	walker β_2	distance β_3	distance^2 β_4	initiator β_5	initiator : age β_6	initiator : walker β_7	initiator : distance β_8	initiator : distance^2 β_9
Analysis1										
1	4805.62 (103.63)	0.043 (0.015)	–	1.84 (0.244)	-0.796 (0.126)					
2	4808.07 (103.06)	0.057 (0.022)	-0.078 (0.087)	1.84 (0.240)	-0.794 (0.124)					
3	4810.96 (106.72)	–	0.093 (0.060)	1.82 (0.237)	-0.788 (0.123)					
Analysis2										
1	6686.06 (79.24)	-0.037 (0.019)	–	1.38 (0.258)	-0.830 (0.132)	-2.41 (0.334)	0.148 (0.022)	–	0.936 (0.104)	–
2	6962.95 (81.07)	-0.037 (0.025)	-0.020 (0.091)	1.29 (0.247)	-0.819 (0.130)	-2.42 (0.344)	0.146 (0.022)	–	0.987 (0.107)	–
3	6964.44 (81.22)	-0.039 (0.028)	-0.010 (0.111)	1.29 (0.249)	-0.817 (0.130)	-2.45 (0.412)	0.150 (0.032)	-0.020 (0.128)	0.989 (0.106)	–
Analysis3										
1	2424.38 (39.79)	0.166 (0.026)	–	1.06 (0.126)	–					
2	2425.35 (39.40)	0.170 (0.039)	-0.016 (0.153)	0.894 (0.450)	0.101 (0.240)					
3	2425.83 (39.81)	0.168 (0.039)	-0.012 (0.150)	1.06 (0.125)	–					

Table S5. The posterior distribution of the parameters of the best model in Analysis 3. The mean (EAP) and quantiles (2.5% and 97.5%) of the posterior distribution are shown.

	Parameter	EAP	2.5%	97.5%
Analysis3				
age	β_1	0.166	0.115	0.218
distance	β_3	1.06	0.821	1.32

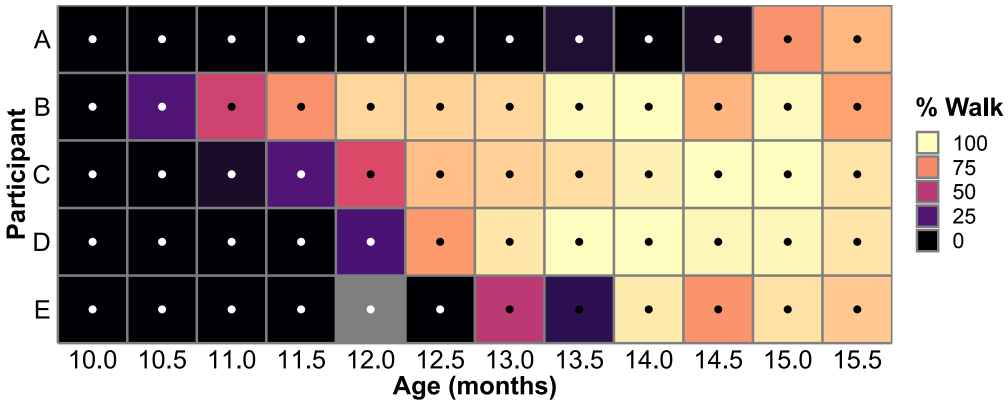

Figure S1. Each infant's locomotor status during the longitudinal observation. The rows indicate unique individuals. The x-axis represents age in months of each infant on the observation day. The colour of the tile represents the proportion of the infant's walking time to the sum of walking time and crawling time. The colour of the dots indicates whether the infant's locomotor status was crawler (white) or walker (black). Note that there was one observation day (when infant E was 12 months old) on which we could not collect data (missing data).

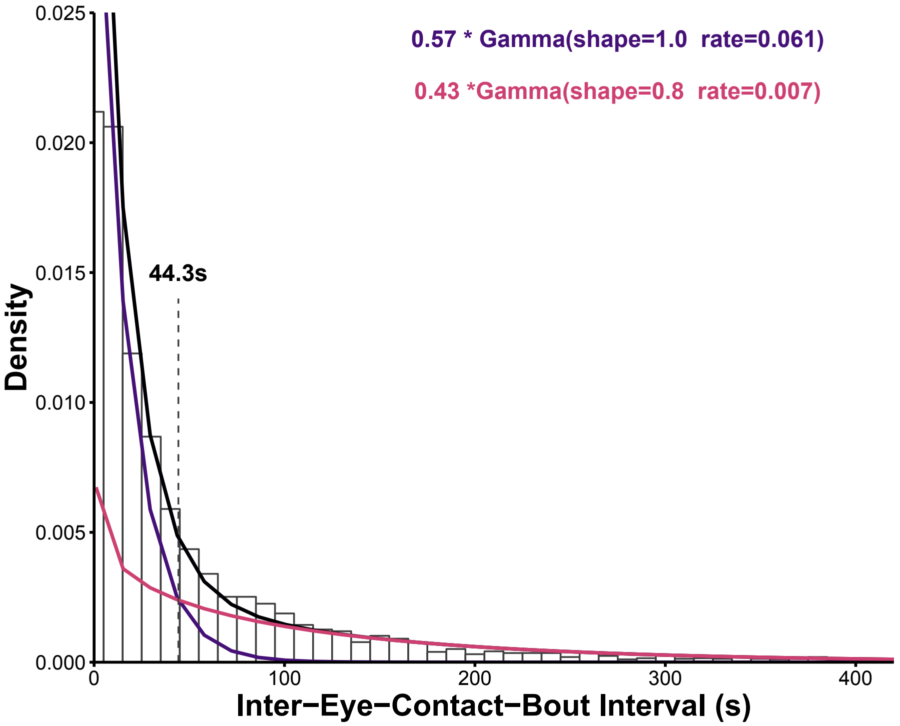

Figure S2. Distribution of the inter-eye-contact-bout intervals (IEIs), the fitted curves of the combination of the 2 gamma distribution model (black line) and its 2 components of gamma distribution curves representing IEIs in the same eye contact session (EC session) (purple line) and IEIs between adjacent EC sessions (pink line). We determined the IEI criteria (dashed vertical line) to define the EC session from the estimated parameters of the 2-process model.

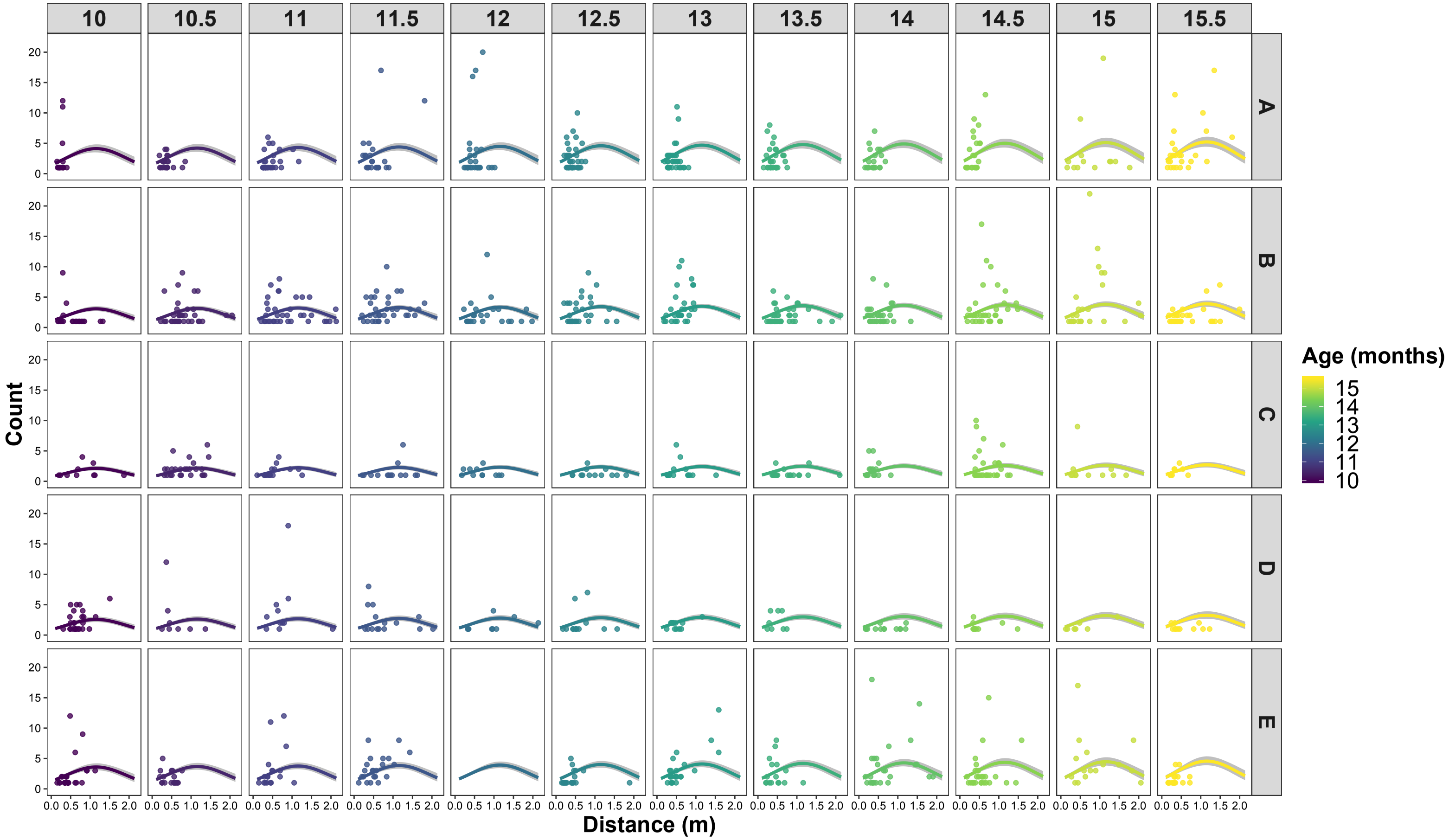

Figure S3. Predictions of the best model in Analysis 1. The observed data (coloured dots), posterior mean (coloured lines) and 95% credible interval (grey areas) of the number of EC bouts in each EC session are shown in each subplot. The colour of the dots and lines represents the infant's age in months. All subplots are for each participant for each observation day. The subplots on the row dimension represent the longitudinal change in the gaze communication of each infant-parent dyad. The subplots on the column dimension represent individual differences in the gaze communication of infants that are the same age in months.

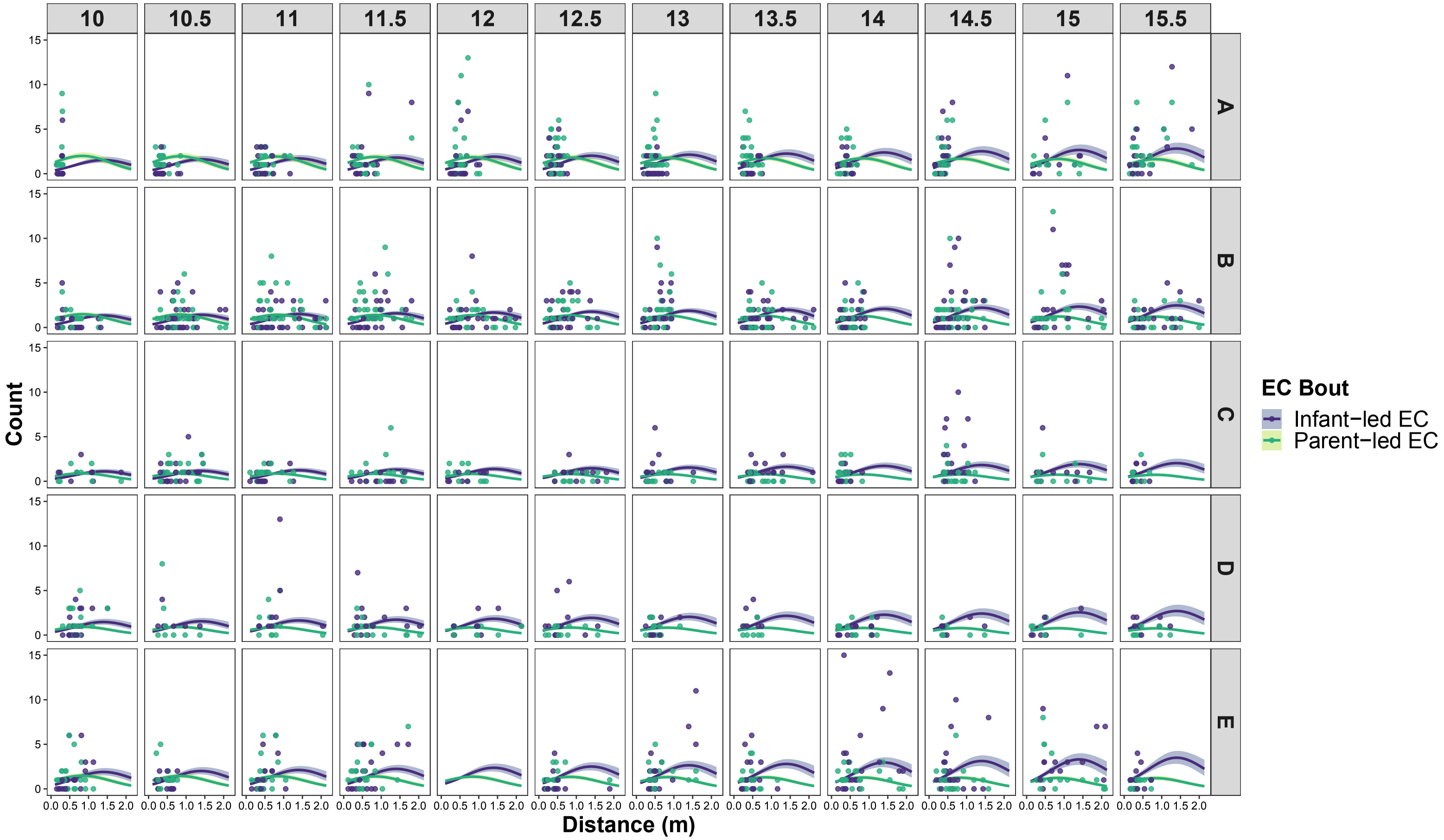

Figure S4. Predictions of the best model in Analysis 2. The observed data (coloured dots), posterior mean (coloured lines) and 95% credible interval (coloured areas) of the number of infant-led EC bouts (blue) and parent-led EC bouts (green) in each EC session are shown in each subplot. All subplots are for each participant and each observation day. The subplots on the row dimension represent the longitudinal change in the gaze communication of each infant-parent dyad. The subplots on the column dimension represent individual differences in the gaze communication of infants that are the same age in months.

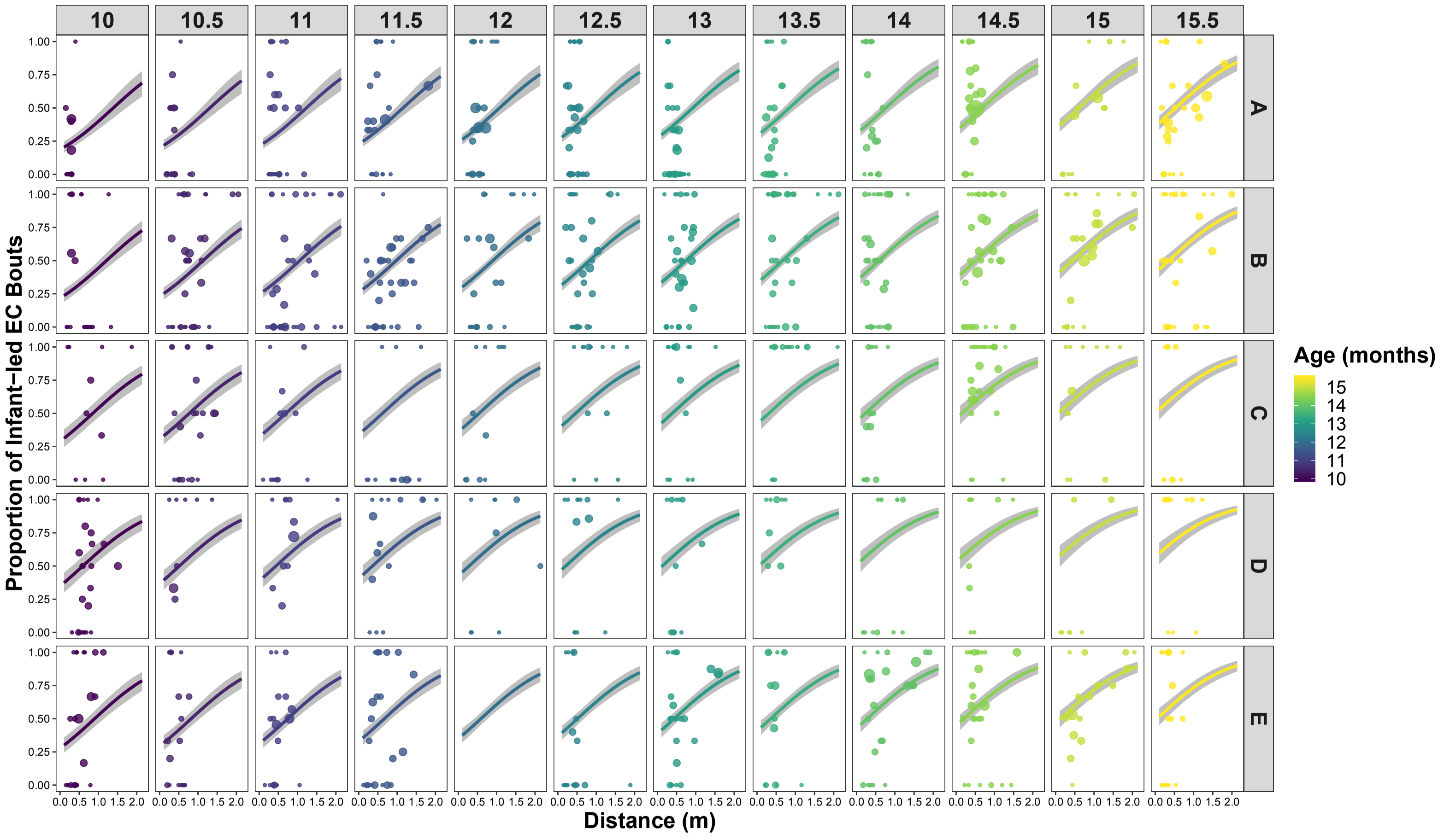

Figure S5. Predictions of the best model in Analysis 3. The observed data (coloured dots), posterior mean (coloured lines) and 95% credible interval (grey areas) of the proportion of infant-led EC bouts in each EC session are shown in each subplot. The colour of dots and lines represents the infant's age in months, and the size of dots represents the total number of EC bouts in each EC session. All subplots are for each participant and each observation day. The subplots on the row dimension represent the longitudinal change in the gaze communication of each infant-parent dyad. The subplots on the column dimension represent individual differences in the gaze communication of infants that are the same age in months.
